# The Main Immune Markers of Ligamentum Flavum Hypertrophy: A Narrative Literature Review

**DOI:** 10.3390/ijms27156690

**Published:** 2026-07-27

**Authors:** Domantas Tamasauskas, Laura Tamasauskiene, Brigita Gradauskiene

**Affiliations:** 1 Department of Immunology and Allergology, Lithuanian University of Health Sciences, Eiveniu Str. 2, LT-50009 Kaunas, Lithuania; domantas.tamasauskas@lsmu.lt (D.T.);; 2 Department of Neurosurgery, Lithuanian University of Health Sciences, Eiveniu Str. 2, LT-50009 Kaunas, Lithuania; 3 Laboratory of Immunology, Department of Immunology and Allergology, Lithuanian University of Health Sciences, Eiveniu Str. 2, LT-50009 Kaunas, Lithuania

**Keywords:** ligamentum flavum, hypertrophy, immune markers, cytokines, inflammation, fibrosis, oxidative stress

## Abstract

LF hypertrophy contributes to the narrowing and compression of neural structures, impairing limb function. It is a leading cause of spinal stenosis, particularly in the lumbar region. About 20% of those over 60 show lumbar stenosis on imaging, but over 80% have no symptoms. Surgery is the main treatment option and is only recommended for symptomatic cases. The exact cause of LF hypertrophy remains unclear, though chronic inflammation, fibrosis and oxidative stress are implicated. Research consistently shows that increased cytokines and growth factors drive extracellular matrix remodeling, immune cell infiltration, and fibroblast and osteoblast activation. The aim of this article is to briefly review the role of the main immune markers that are involved in the pathogenesis of LF hypertrophy. This narrative review is based on a PubMed literature search and includes 23 original articles published between 2019 and 2026.

## 1. Introduction

The ligamentum flavum (LF) connects adjacent vertebrae and plays a crucial role in maintaining spinal stability and protecting the spinal cord [1,2]. The LF is an elastic structure composed of elastin (80%) and collagen (20%) fibers [1]. The thickness of the normal lumbar LF is approximately 2 to 4 mm but can be as high as 7 to 15 mm when LF hypertrophy occurs [2]. LF hypertrophy contributes to narrowing and compression of the spinal canal, intervertebral foramina, and nerve root canals. This compression adversely affects the motor and sensory functions of the extremities. LF hypertrophy is one of the most frequent causes of spinal stenosis [1,2]. The incidence of LF hypertrophy has increased substantially over the past decade, particularly among older adults [3].

Spinal stenosis most commonly affects the lumbar region. Lumbar spinal stenosis is characterized by narrowing of the lumbar spinal canal and/or the neural foramina, with compression of the spinal nerve roots [4]. Lumbar spinal stenosis usually develops with older age and is a degenerative condition. Patients with this disease may experience lower back and leg pain, numbness in the lower limbs and intermittent claudication [4,5]. Approximately 20% of people older than 60 years have imaging evidence of lumbar spinal stenosis, and more than 80% of them are asymptomatic [4]. Treatment is recommended only when spinal stenosis is symptomatic. This indicates that LF hypertrophy may continue to progress even without symptoms.

The exact pathogenetic mechanism of LF hypertrophy is unclear. However, research suggests it involves fibrotic changes characterized by a loss of elastic fibers and an increase in collagen fibers as hypertrophy progresses [1,5]. The main risk factors for LF hypertrophy are older age and mechanical stress [5,6]. Abnormal mechanical stress can induce micro-damage of the LF tissue, which leads to chronic inflammation and promotes LF hypertrophy development [5]. Moreover, the development of fibrosis in LF tissue involves multiple cytokines [5,7,8]. A thorough understanding of the mechanisms leading to LF hypertrophy is essential for developing treatments and preventing spinal stenosis progression. The aim of this article is to briefly review the role of the main immune markers that are involved in the pathogenesis of LF hypertrophy.

## 2. Criteria for Articles Involved in This Review

### 2.1. Measured Outcomes

The primary focus was on immune markers involved in the pathogenesis of LF hypertrophy. The primary outcome measure was to classify the main immune markers and their mechanism of action in LF hypertrophy. Secondary outcomes of interest included: the relation between ligamentum flavum thickness and immune markers, the relation between immune markers and severity of symptoms of lumbar stenosis, and the relation between local (LF) and systemic (blood) immune markers. Data on the number and age of participants were also collected where available.

### 2.2. Eligibility Criteria

English-language observational studies (cross-sectional) and experimental studies were included if they analyzed the role of immune markers in patients with spinal stenosis caused by LF hypertrophy and were published within the period of 2019–2026.

### 2.3. Search Strategy

Studies were searched for in the PubMed database. The Medical Subject Heading (MeSH) terms of “spinal stenosis” or “ligamentum flavum hypertrophy” and “cytokines” or “immune markers” or “immune cells” or “inflammation” were used. In addition, combined text words of (spinal stenosis AND cytokines) OR (spinal stenosis AND immune markers) OR (spinal stenosis AND immune cells) OR (spinal stenosis AND inflammation) OR (ligamentum flavum AND cytokines) OR (ligamentum flavum AND immune markers) OR (ligamentum flavum AND immune cells) OR (ligamentum flavum AND inflammation) were used to find relevant studies.

A total of 94 articles were found. Of these articles, 52 were eliminated after an initial screening of their titles and abstracts (reviews, animal research, no access to full text, or unrelated outcome). The full texts of the 42 remaining articles were assessed in detail. Nineteen articles were rejected (unrelated outcome). Finally, 23 articles were included in the review.

## 3. Results and Discussion

### 3.1. Cytokines in LF Hypertrophy Pathogenesis

LF hypertrophy, a key contributor to lumbar spinal stenosis, is increasingly understood as a chronic inflammatory and fibrotic condition. Moreover, oxidative stress plays a significant role in the pathogenesis of this disorder. A consistent theme across the scientific literature is the upregulation of several cytokines and growth factors that orchestrate extracellular matrix (ECM) remodeling, immune cell infiltration, fibroblast and osteoblast activation and the production of reactive oxygen species (ROS). The immune system mediators that are involved in the pathogenesis of LF hypertrophy can be broadly categorized into pro-inflammatory, pro-fibrotic, and anti-inflammatory cytokines, with significant crosstalk among their pathways.

### 3.2. Pro-Inflammatory Cytokines: TNF-α, IL-1β, IL-6, IL-17

TNF-α is one of the most frequently implicated cytokines in LF hypertrophy. Multiple studies have demonstrated elevated tumor necrosis factor (TNF)-α levels in hypertrophic LF tissue compared to controls [9,10,11]. TNF-α promotes inflammation by activating the NF-κB and p38 MAPK signaling pathways, leading to the upregulation of other cytokines such as interleukin (IL)-6 and fibrogenic mediators like transforming growth factor-beta 1 (TGF-β1) [12]. It also contributes to ROS generation, which further amplifies fibrotic responses. A study performed by Ito et al. showed high protein expression of TNF-α and the marker of oxidative stress 8-hydroxy-2′-deoxyguanosine (8-OHdG) in hypertrophied LF [10]. Moreover, a correlation between the positive cell numbers for TNF-α and 8-OHdG was found. This study also revealed that H_2_O_2_, buthionine sulfoximine (BSO), and TNF-α treatment significantly increased intracellular ROS levels in LF cells compared with the control [10]. BSO treatment induced type I and type III collagen and TNF-α mRNA expression. Takeda et al. showed that mRNAs for collagen types I, III, VI, V, VIII, XII, and XV were highly expressed in LF samples. Protein expression of collagen types I, III, V, VI, and VIII was observed in both non-hypertrophied and hypertrophied LF [13]. However, collagen types I, III and VI were associated with LF hypertrophy. Moreover, the mRNA expressions of almost all collagens were significantly up-regulated by treatment with both TNF-α and IL-1β in the LF cells [13].

Nagai et al. explored the role of the oxidized low-density lipoprotein (oxLDL)/lectin-type oxidized LDL receptor-1 (LOX-1) axis in the pathogenesis of LF hypertrophy, providing a mechanistic link between atherosclerosis-related inflammation and lumbar spinal canal stenosis [14]. LF specimens were obtained from patients undergoing posterior lumbar surgery, and LOX-1 expression was assessed by immunohistochemistry, showing a weak but significant correlation between the number of LOX-1-positive cells and the LF cross-sectional area measured on preoperative magnetic resonance imaging (MRI). In vitro experiments using primary human LF cells demonstrated that the pro-inflammatory cytokines TNF-α and IL-1β significantly upregulated LOX-1 expression at both the mRNA and protein levels, indicating that inflammatory signaling enhances oxLDL responsiveness in LF tissue [14]. Exposure to oxLDL induced the expression of LF hypertrophy-associated markers, including type I collagen, type III collagen, and cyclooxygenase-2 (COX-2), accompanied by activation of the MAPK and NF-κB signaling pathways [14].

Interleukin (IL)-1β is a pro-inflammatory cytokine significantly upregulated in hypertrophied LF tissue. In a recent study using immunohistochemistry and ELISA, IL-1β levels were found to be markedly higher in hypertrophic LF samples compared to non-hypertrophic samples, indicating its involvement in the local inflammatory response contributing to LF thickening [9]. As mentioned previously, this cytokine can increase the production of collagens in the LF [13]. Hsu et al. conducted an original human study with the aim to investigate how pro-inflammatory cytokines influence the lineage commitment of progenitor cells derived from degenerative intervertebral disc and LF tissues in patients with degenerative spondylolisthesis and lumbar spinal stenosis [15]. Cells from intervertebral discs and LF showed mesenchymal stem cell-like traits, with a fibroblast-like shape; expression of CD44, CD90, and CD105; and strong potential for osteogenic and adipogenic differentiation in vitro. Functional experiments demonstrated that cytokine exposure markedly altered lineage commitment: IL-1β consistently promoted osteogenic differentiation while suppressing adipogenesis in both intervertebral disc- and LF-derived cells [15].

IL-6 is both an inflammatory and fibrogenic cytokine. Some studies showed higher IL-6 mRNA and IL-6 levels in hypertrophic LF tissue than in normal LF tissue [16]. Yayama et al. investigated cytokine expression profiles in cervical ossification of the posterior longitudinal ligament (OPLL) to elucidate the immunological mechanisms associated with progressive ossification [17]. This condition has a pathogenesis similar to that of LF hypertrophy. Ligamentous tissues were obtained from patients undergoing surgery for cervical OPLL and compared with control samples from patients with cervical spondylotic myelopathy or radiculopathy without OPLL. Histological and immunohistochemical analyses were combined with cytokine profiling of primary ligament-derived cells investigating 27 inflammatory cytokines and growth factors [17]. The analysis demonstrated significantly increased levels of IL-6, IL-1α, basic fibroblast growth factor (bFGF), and RANTES in OPLL specimens compared with controls [17]. The IL-6 level in the cell culture supernatant of cells from LF with thoracic ossification was significantly higher compared with that for LF from healthy subjects [18]. Moreover, IL-6 stimulation activated the expression of osteoblastic factors and the expression of cell cycle-related genes such as cell proliferation factors cyclin D1 and cyclin C [18]. IL-6 is significantly upregulated in LF hypertrophy and acts through the JAK/STAT3 and p38 MAPK pathways, promoting fibroblast and osteoblast activation and ECM component production. IL-6 acts indirectly on osteoclastogenesis by stimulating the release of RANKL by cells within bone tissues, such as osteoblasts. Leptin, an adipokine elevated in LF hypertrophy, has been shown to contribute to fibrosis and hypertrophy by activating IL-6 expression in LF fibroblasts. This process promotes chronic inflammation and collagen production, linking metabolic status with fibrotic remodeling of the LF [16,17]. IL-6 is associated with inflammation, mechanotransduction, and osteo-fibrotic remodeling in various spinal ligament disorders.

Different studies highlight that pro-inflammatory cytokines interact synergistically, with TNF-α amplifying IL-6 and IL-1β expression through NF-κB activation, creating a persistent inflammatory microenvironment that accelerates extracellular matrix turnover. Lee et al. conducted an original human study demonstrating that hypertrophic LF in lumbar spinal stenosis is strongly associated with increased expression of secreted protein acidic and rich in cysteine (SPARC), a matricellular protein implicated in fibrotic remodeling [19]. Hypertrophic LF specimens obtained during decompressive laminectomy were compared with non-hypertrophic LF from age- and sex- matched disc herniation controls, showing significantly greater LF thickness on MRI and markedly higher fibrosis scores on Masson’s trichrome staining. Immunohistochemical and quantitative PCR analyses revealed substantial upregulation of SPARC, collagen III, and α-smooth muscle actin, alongside reduced elastin expression, indicating advanced fibrotic transformation of LF tissue [19]. Primary LF cells isolated from hypertrophic samples exhibited elevated inflammatory and fibrotic markers, including IL-6, IL-1β, and TGF-β, compared with non-hypertrophic samples. IL-6 stimulation directly induced SPARC expression together with collagen I, collagen III, connective tissue growth factor, and pro-inflammatory cytokines [19].

Yabu et al. demonstrated that mechanical stress is a central driver of molecular remodeling in the LF, identifying periostin as a key mechanosensitive mediator contributing to hypertrophy [20]. Using a long-term (1-year) mechanical stress model combined with integrated analysis of three independent transcriptomic datasets, the authors found that periostin expression consistently increased in response to sustained mechanical loading [20]. This finding was validated in human LF specimens, where periostin, biglycan and HAPLN1 were significantly upregulated in hypertrophied tissue, and periostin levels strongly correlated with LF thickness. In vitro experiments further showed that both fluid shear stress and TGF-β1 stimulation increased periostin expression alongside fibrotic markers (α-SMA, Col1a1) and the inflammatory cytokine IL-6 [20]. Functional inhibition assays revealed that suppressing periostin reduced the mechanical stress-induced expression of fibrotic and inflammatory mediators, indicating that periostin partly acts through the integrin and NF-κB signaling pathways [20]. Taken together, these findings suggest that periostin acts as a mechanotransduction and pro-inflammatory regulator in LF hypertrophy, linking mechanical loading to IL-6-driven fibrosis.

An interesting study was performed which evaluated the biochemical differences in the LF between smokers and non-smokers [21]. The results showed that smokers exhibited significantly higher levels of IL-1β, IL-6, and TNF-α compared to non-smokers, which means that smoking contributes to systemic inflammation and oxidative stress, leading to biochemical changes that may drive LF hypertrophy [21].

Recent findings suggest a potential role for IL-17 in the pathogenesis of LF hypertrophy. Bioinformatics analysis of LF hypertrophic tissue revealed enrichment in Th17 cells (11). However, direct quantification of IL-17 expression is not consistently reported across studies. A study performed by Lin et al. showed that IL-17A was elevated in cases of thoracic ossification of the LF tissue compared with normal LF [22]. IL-17A promoted the proliferation and osteogenic differentiation of LF cells by regulating β-catenin signaling [22]. These findings suggest a regulatory role for IL-17 in immune cell-mediated inflammation and matrix remodeling, warranting further validation.

### 3.3. Fibrogenic Cytokines: TGF-β1 and MIF

TGF-β1 is considered a central cytokine driving fibrosis in LF hypertrophy. Human LF fibroblasts can be stimulated by mechanical and oxidative stress, leading to overproduction of TGFβ1, which in turn facilitates the deposition of ECM [5]. TGFβ1 stimulates the Smad2/3 and MAPK pathways, promoting the differentiation of fibroblasts into α-SMA-positive myofibroblasts—a key event in LF thickening and stiffness [5,23]. TGF-β1 also upregulates the synthesis of types I and III collagen, vimentin, β-catenin and connective tissue growth factor (CTGF), reinforcing the fibrotic ECM environment [5,19,23]. Increased expression of TGF-β1 has been confirmed by both immunohistochemistry and ELISA in multiple human studies [11,12,24,25]. Recent analyses further demonstrate that sustained TGF-β1 signaling promotes fibroblast-to-myofibroblast transition while simultaneously suppressing elastin synthesis, shifting the ligament toward a mechanically stiffer, collagen-dominant phenotype. Goto et al. presented significant mechanistic evidence that connects TGF-β signaling with IL-6-mediated inflammatory activation in human LF-derived cells [26]. Using primary LF cells obtained from patients undergoing decompression surgery for lumbar spinal stenosis, the authors demonstrated that TGF-β stimulation induces IL-6 secretion in a clearly dose- and time-dependent manner. Western blot analyses further revealed that TGF-β rapidly activates both p38 and p44/42 MAP kinases, with p44/42 phosphorylation occurring within minutes and p38 phosphorylation peaking at 2–3 h [26]. Pharmacologic inhibition experiments showed that blocking either p38 or p44/42 markedly suppressed TGF-β-induced IL-6 secretion and IL-6 mRNA expression, indicating that both MAPK pathways are required for IL-6 upregulation. Because IL-6 is elevated in hypertrophied LF and correlates with pain and fibrosis in LSS, these findings provide a direct molecular link between TGF-β signaling, which is often increased in mechanically stressed or degenerative LF tissue, and inflammatory amplification via IL-6 [26]. Overall, the study demonstrated that TGF-β stimulates IL-6 protein secretion and gene expression in LF cells by activating the p38 or p44/42 MAP kinase pathway. Zhao et al. identified thrombospondin-1 (THBS1) as a central mechanosensitive mediator linking mechanical stress to fibrotic remodeling in LF hypertrophy [27]. Using human LF specimens obtained during decompressive surgery, the authors demonstrated that hypertrophic LF exhibited marked elastin degradation, excessive collagen deposition, and significantly increased expression of THBS1, TGF-β1, α-smooth muscle actin, and COL1A2 at both the transcript and protein levels, with THBS1 expression correlating with ligament thickness measured by MRI [27]. Proteomic profiling (iTRAQ) and single-cell RNA sequencing of clinical LF samples further confirmed robust upregulation of THBS1 predominantly within fibroblast and myofibroblast populations, together with enrichment of extracellular matrix organization and TGF-β-related pathways. In vitro experiments using primary human LF fibroblasts showed that cyclic mechanical stretching induced time-dependent increases in THBS1 and activated TGF-β1/Smad3 signaling, resulting in enhanced expression of fibrotic markers [27]. Importantly, pharmacological inhibition of THBS1 attenuated mechanical stress-induced TGF-β1 activation and downstream fibrosis-associated gene expression, supporting a causal role for THBS1 in mechanotransduction-driven fibrogenesis [27]. This study provides strong human-based evidence that THBS1 functions as an upstream activator of TGF-β1/Smad3 signaling in LF hypertrophy, linking chronic mechanical overload to progressive fibrosis. Liu et al. identified clusterin as an endogenous negative regulator of mechanical stress-induced LF hypertrophy, providing mechanistic insight into feedback control of TGF-β1-driven fibrosis in the human LF [28]. Using LF specimens obtained from patients with lumbar spinal canal stenosis and from non-hypertrophic controls, the authors applied iTRAQ-based proteomic profiling and demonstrated that clusterin was among the most strongly upregulated proteins in hypertrophic LF, with expression levels correlating with ligament thickness and histological fibrosis severity. Immunohistochemical, transcriptomic, and protein analyses confirmed concomitant upregulation of clusterin, TGF-β1, α-smooth muscle actin, and COL1A2 in hypertrophic LF tissue, indicating active myofibroblast differentiation [28]. Functional in vitro experiments using primary human LF fibroblasts showed that cyclic mechanical stretch and TGF-β1 stimulation induced clusterin expression in a time-dependent manner. Importantly, exogenous clusterin attenuated mechanical stress and TGF-β1 induced fibrotic responses by suppressing SMAD3 phosphorylation and nuclear translocation through competitive interaction with the TGF-β type I receptor ALK5. These results demonstrate that clusterin functions as a mechanosensitive antifibrotic modulator, regulating TGF-β1/SMAD3 signaling under conditions of prolonged mechanical stress.

Macrophage migration inhibitory factor (MIF) has recently been identified as a novel contributor to LF hypertrophy [25]. MIF induces proliferation of LF fibroblasts via the Src kinase signaling pathway, and it upregulates TGF-β1, MMP-13, and collagen synthesis [25]. Its role highlights the interplay between inflammation and fibrosis, as well as the emerging recognition of non-traditional cytokines in spinal ligament pathology.

### 3.4. Anti-Inflammatory Cytokines: IL-10

IL-10 is an anti-inflammatory cytokine that modulates the immune response and limits excessive fibrosis. Its upregulation in some LF hypertrophy models may reflect a feedback mechanism attempting to resolve chronic inflammation [16]. However, this response is typically insufficient to prevent disease progression. Although IL-10 increases in some samples, it does not effectively counteract persistent TNF-α and IL-6 activity, indicating that cytokine imbalance characterizes LF hypertrophy.

### 3.5. Immune Cell Infiltration and Cytokine Crosstalk

Immunological studies confirm the infiltration of immune cells, particularly M2-polarized macrophages, into hypertrophic LF tissue. Li et al. conducted an original human study to characterize macrophage polarization and macrophage-related molecular signatures in hypertrophic LF associated with lumbar spinal stenosis. Ligamentum flavum specimens were obtained from patients with hypertrophic LF and compared with non-hypertrophic LF specimens from disc herniation controls, with LF thickness quantified using preoperative MRI and fibrosis evaluated histologically. Hypertrophic LF exhibited marked structural degeneration, including disrupted elastic fibers, excessive collagen deposition, and increased cellular infiltration, accompanied by significant upregulation of the extracellular matrix components aggrecan, collagen I, and collagen II. Immunohistochemical and ELISA analyses demonstrated elevated levels of the pro-inflammatory cytokines TNF-α and IL-1β, as well as the pro-fibrotic mediator TGF-β1, in hypertrophic LF compared with controls. Notably, macrophage profiling revealed predominant infiltration of CD206^+^ M2-type macrophages in hypertrophic LF, whereas the M1 macrophage marker CCR7 was absent, suggesting that late-stage hypertrophic LF is characterized by a pro-fibrotic macrophage phenotype rather than active classical inflammation. In addition, increased expression of the aging markers p53, p16, and p21 in hypertrophic LF linked chronic inflammation, extracellular matrix remodeling, and cellular aging within the ligament. These findings provide direct human evidence that M2-polarized macrophages, together with persistent inflammatory and fibrotic signaling, contribute to LF hypertrophy. However, Qu et al. found that the concentrations of TNF-α, IL-1β and IL-6 in M1 macrophage supernatants were greater than those in M2 macrophages, and greater levels of these cytokine receptors were observed in LF cells induced with M1 than those with M2 [29]. Osteogenic differentiation of LF cells induced by M1 decreased only after IL-6 was neutralized [29]. These findings suggest that M1 macrophage-derived IL-6 promotes the osteogenic differentiation of LF cells. While M2 macrophages are prevalent in hypertrophic LF, TNF-α and IL-1β suggest prior M1 activity or mixed macrophage phenotypes in chronic disease.

A new and relevant study was performed by Fei et al. where scientists applied an integrated single-cell and multi-omics strategy to delineate the cellular and molecular landscape of LF hypertrophy, revealing ferroptosis as a previously unrecognized driver of fibrotic progression [30]. Using scRNA-seq profiling of human LF tissues from non-LF hypertrophy and LF hypertrophy patients, the authors identified marked expansion of fibroblasts, myofibroblasts, and macrophages, alongside profound alterations in extracellular matrix organization. Transmission electron microscopy and ferroptosis-associated gene signatures demonstrated increased lipid peroxidation, iron accumulation, and mitochondrial damage in hypertrophic LF [30]. These findings were further supported by reduced expression of anti-ferroptotic regulators (GPX4, FTL) and increased levels of pro-ferroptotic ACSL4 at both the protein and transcript levels. Subcluster analysis uncovered a fibroblast population with a high ferroptosis score, which exhibited enhanced fibrotic activity. Notably, multi-omics integration identified a disease-specific subset of SPP1^+^ macrophages whose ligand–receptor interactions (particularly the SPP1–CD44 axis) strongly targeted high-ferroptosis fibroblasts, facilitating their activation and matrix deposition (30). RNA-seq deconvolution and Mendelian randomization further supported a causal link between iron metabolism dysregulation and spinal stenosis. Collectively, this study established ferroptosis-mediated macrophage–fibroblast crosstalk as a central mechanism in the pathogenesis of LF hypertrophy and highlighted SPP1^+^ macrophages and ferroptosis pathways as promising diagnostic and therapeutic targets.

Additional evidence indicates that macrophage-driven remodeling is accompanied by T-cell infiltration, particularly with Th17 cells, which contribute IL-17A, further stimulating fibroblast proliferation and osteogenic differentiation in LF tissue [8,11].

**Figure 1:**
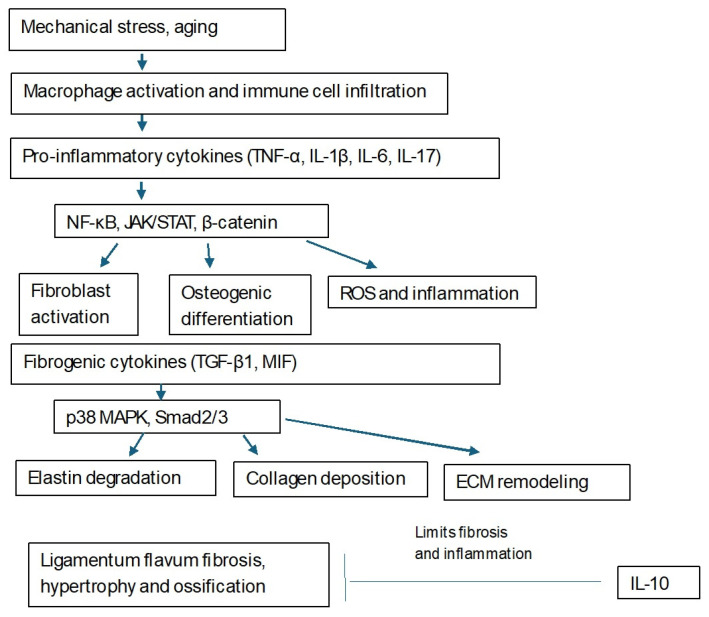
Cytokines involved in LF hypertrophy.

A summary of the key cytokines involved in LF hypertrophy is presented in Table 1 and Figure 1.

## 4. Conclusions

Unlike previous reviews focusing primarily on mechanical or degenerative aspects of LF hypertrophy, this narrative review emphasizes immune-mediated mechanisms and integrates cytokines, immune cells, and mechanotransduction pathways into a unified immunopathological framework.

LF hypertrophy is a multifactorial process driven by chronic inflammation and fibrosis, with a central role played by cytokines such as TNF-α, IL-1β, IL-6, and TGF-β1. These mediators orchestrate extracellular matrix remodeling, immune cell recruitment, oxidative stress, and fibroblast and osteoblast activation. While multiple cytokines have been consistently identified as contributors to LF hypertrophy, the evidence is still evolving. Most studies rely on human LF tissue samples from surgical patients, and relatively few include mechanistic or interventional in vitro or in vivo models. Moreover, human studies lack information about the relation between cytokines in LF and clinical symptoms of spinal stenosis. It is not clear whether an increase in cytokines in the LF correlates with the cytokine levels in blood. There remains a lack of consensus on the temporal sequence of cytokine expression, particularly regarding IL-10 and IL-17, which are likely context- and stage-dependent. The predominance of M2 macrophages suggests a shift from inflammation toward fibrosis, though further studies are needed to determine whether this transition is universal or variable. Importantly, the immune microenvironment of the LF may offer a future target for anti-fibrotic therapies, particularly those modulating the TGF-β1 and IL-6 pathways. Better understanding of the cytokine profile in LF hypertrophy may facilitate the development of targeted therapies to prevent or slow the progression of spinal stenosis.

## Figures and Tables

**Table 1:** Summary of cytokines involved in pathogenesis of LF hypertrophy. Abbreviations: IHC—immunohistochemistry; ELISA—enzyme-linked immunosorbent assay; qRT-PCR—quantitative real-time polymerase chain reaction; WB—Western blot; HLF—hypertrophy of the ligamentum flavum; ROS—reactive oxygen species.

Cytokine	Experimental Method(s)	Finding(s)
IL-1β	ELISA, IHC	Elevated in HLF; local inflammatory response; increases production of collagen; promotes osteogenic differentiation.
TNF-α	IHC, qRT-PCR, WB, flow cytometry	Elevated in HLF; promotes inflammation by activating the NF-κB and p38 MAPK signaling pathways; upregulation of IL-6 and TGF-β1; contributes to ROS generation; amplifies fibrotic responses.
IL-6	IHC, qRT-PCR, WB	Elevated in HLF; linked to TGF-β activity and involved in JAK/STAT and p38 MAPK signaling; promotes inflammation, fibroblast and osteoblast activation, and ECM component production.
IL-17	qRT-PCR, WB	Elevated in HLF; promotes inflammation and proliferation and osteogenic differentiation of LF cells by regulating β-catenin signaling.
TGF-β1	IHC, qRT-PCR, WB	Elevated in HLF; promotes collagen synthesis and fibrosis through Smad2/3 and MAPK pathways.
MIF	IHC, ELISA	Promotes fibrosis via the Src kinase signaling pathway and upregulates TGF-β1 and MMP-13.
IL-10	IHC, qRT-PCR	Increased in HLF; potentially anti-inflammatory—modulates the immune response and limits excessive fibrosis.

## Data Availability

No new data were created or analyzed in this study. Data sharing is not applicable to this article.

## Abbreviations

LF	ligamentum flavum
ECM	extracellular matrix
ROS	reactive oxygen species
TNF	tumor necrosis factor
TGF-β1	transforming growth factor-beta 1
8-OHdG	8-hydroxy-2′-deoxyguanosine
BSO	buthionine sulfoximine
oxLDL	oxidized low-density lipoprotein
LOX-1	lectin-type oxidized LDL receptor-1
MRI	magnetic resonance imaging
COX-2	cyclooxygenase-2
IL	interleukin
OPLL	ossification of the posterior longitudinal ligament
bFGF	basic fibroblast growth factor
SPARC	secreted protein acidic and rich in cysteine
THBS1	thrombospondin-1
MIF	migration inhibitory factor
